# A simplified, robust, and streamlined procedure for the production of *C. elegans *transgenes via recombineering

**DOI:** 10.1186/1471-213X-8-119

**Published:** 2008-12-30

**Authors:** Yue Zhang, Lindsey Nash, Alfred L Fisher

**Affiliations:** 1 University of Pittsburgh, Department of Medicine, Division of Geriatric Medicine and Pittsburgh Institute for Neurodegenerative Diseases, Pittsburgh, PA 15260, USA; 2 Dept. of Radiation Oncology, BIDMC, Boston, MA 02215, USA

## Abstract

**Background:**

The nematode *Caenorhabditis elegans *has emerged as a powerful system to study biologic questions ranging from development to aging. The generation of transgenic animals is an important experimental tool and allows use of GFP fusion proteins to study the expression of genes of interest or generation of epitope tagged versions of specific genes. Transgenes are often generated by placing a promoter upstream of a reporter gene or cDNA. This often produces a representative expression pattern, but important exceptions have been observed. To better capture the genuine expression pattern and timing, several investigators have modified large pieces of DNA carried by BACs or fosmids for use in the construction of transgenic animals via recombineering. However, these techniques are not in widespread use despite the advantages when compared to traditional approaches. Additionally, some groups have encountered problems with employing these techniques. Hence, we sought identify ways to improve the simplicity and reliability of the procedure.

**Results:**

We describe here several important modifications we have made to existing protocols to make the procedure simpler and more robust. Among these are the use of *galK *gene as a selection marker for both the positive and negative selection steps in recombineering, the use of R6K based plasmids which eliminate the need for extensive PCR product purification, a means to integrate the *unc-119 *marker on to the fosmid backbone, and placement of homology arms to commonly used GFP and TAP fusion genes flanking the *galK *cassette which reduces the cost of oligos by 50%.

**Conclusion:**

We have made several significant changes that allow the production of *C. elegans *transgenes from a commercially available fosmid library in a robust and streamlined manner. These changes make the technique more attractive especially to small academic labs unfamiliar with recombineering.

## Background

The generation of transgenic animals in model systems such as *Caenorhabditis elegans *has created dramatic changes in the ability of researchers to approach biologic questions [1]. It is relatively straightforward to create and use a transgenic animal to investigate the timing and pattern of gene expression, express epitope-tagged versions of genes, or test the effects of gene mis-expression.

Traditionally, constructs used for creation of a GFP fusion gene or epitope-tagged version of a gene were drawn from regions of cloned genomic DNA which were known to rescue mutants of the specific gene [1]. As the availability of RNAi, microarrays, and other approaches which are based upon the worm genomic sequence instead of classical mutants has increased, a need to create transgenes in other ways has developed. For these genes, a region 5' of the gene is often isolated via PCR and then used as a promoter [2,3]. This approach has been further simplified by the construction of a large number of vectors by the Fire lab and later by the generation of the *C. elegans *promoterome [1,2,4,5]. However, the decision about the exact length and location of the promoter is more often driven by arbitrary decisions about the size (i.e. 2–3 kb) or convenient restriction sites than sequence analysis. As a result, it is difficult to know if the GFP expression pattern created by a selected promoter reflects the true expression pattern of the gene in vivo [1].

To generate transgenes that are more likely to reflect the in vivo expression timing and pattern, interest in generating transgenics using the larger regions of genomic DNA carried by either fosmids or BACs (bacterial artificial chromosomes) is growing [5-7]. This approach carries the advantage of using 30–300 kb of genomic DNA in the construction of a transgene so it is likely that all or most of the promoter and enhancer elements are included. Additionally since the genomic DNA for the gene is included, additional complexities such as alternative splicing, multiple transcription start sites, and regulation by 3' UTR sequences or microRNA can also occur.

For this approach to become a standard technique within the *C. elegans *community, approaches need to be developed to make the modification of fosmids and BACs quick and robust for the novice. Two recent publications have begun to lay the groundwork for this to occur [6,7]. The first described the modification of *C. elegans *fosmids using homologous recombination in *E. coli *(recombineering) using PCR generated DNA fragments [6]. The creation of a transgene occurred through a two-step procedure where first a *tetA-rpsL *cassette (RT cassette) flanked by homology arms is inserted at the desired location and then in a second step the cassette is replaced by the desired DNA, such as GFP, flanked by the same homology arms. This technique allows the insertion of essentially any sequence at any site in the gene. The second paper described the modification of BACs from the related nematode species *Caenorhabditis briggsae *also by the use of recombineering [7]. In this protocol, a GFP fusion gene is created by inserting GFP at the C-terminus of a gene of interest along with a flanking kanamycin resistance cassette via a single round of recombineering with a PCR product flanked by homology arms. The BAC is also prepared for use as a transgene by insertion of the *unc-119 *gene via recombineering. The addition of the *unc-119 *gene provides an easily visualized selectable marker as this DNA is able to rescue the size, body shape, mobility, and starvation resistance defects present in *unc-119 *mutants [8]. Each approach has strengths and weaknesses for the routine generation of transgenic worms [5].

We have furthered their work by developing a streamlined series of procedures and accompanying vectors to allow the simple and reliable generation of transgenes ready for the transformation of worms by bombardment or microinjection. Specifically, we focused on the use of *C. elegans *genomic DNA cloned into a commercially available fosmid library (Geneservice Ltd., Cambridge, UK). We then developed tools that will allow the robust and efficient generation of either FLAG-GFP or TAP fusion genes by four important changes. First, we utilized the more robust *galK *gene for positive and then negative selection during recombineering [9]. Second, we constructed plasmids for *galK *utilizing the R6K origin which is unable to replicate in the *E. coli *strain used for recombineering and minimizes the amount of processing of the PCR products used [10]. Third, we built homology arms for both GFP and N and C-terminal TAP tags into the *galK *cassette so that a single pair of oligos can be used for the generation of PCR products for each step. The oligos used for recombineering are long and require purification prior to use so the need for one pair instead of two reduces either the labor or cost involved in procuring the oligos. Finally, we developed a protocol for adding the *unc-119 *gene to the fosmid backbone through cre-lox mediated recombination [11]. The final fosmid product can then be used directly for the generation of transgenic nematodes.

## Results

We sought to use the *C. elegans *fosmid library and recombineering for the routine generation of transgenes and subsequent creation of transgenic worms. Two techniques have been described using recombineering in *C. elegans *with one using a BAC library generated from the related nematode species *C. briggsae *while the other used the fosmid library followed by microinjection of the modified plasmids [6,7]. We initially used the protocol described by Dolphin et. al. to successfully generate several *daf-12 *transgenes (data not shown) (Additional files 1, 2, and 3). During this process we identified several ways to optimize and simplify the protocol for recombineering and transgenic animal creation.

The plasmid template used for the generation of the selection/counter-selection cassette by PCR, pBAC-RT, is able to replicate in the SW106 bacterial strain used for recombineering. Consequently, this template needs to be destroyed prior to electroporation either by DpnI digestion or as suggested by Dolphin et. al. through the use of a restriction fragment as the PCR template [6]. As pBAC-RT is constructed on a BAC vector backbone, it is a very low copy number plasmid, and we experienced difficulty in purifying sufficient DNA to digest and gel purify the fragment containing the RT cassette. To address this difficulty, we subcloned the RT cassette from pBAC-RT into the pMOD4 vector (Epicentre Biotechnologies, Madison, WI). pMOD4 uses the R6K replication origin which requires the π protein for replication in *E. coli*. The resulting plasmid pMOD4 RT has the advantages of having a higher copy number than pBAC-RT which simplifies DNA purification and of being unable to replicate in the SW106 bacterial strain. Even when >100 ng. of pMOD4 RT DNA was electroporated into SW106 no tet^R ^colonies were obtained (data not shown). As a result, PCR products used for recombineering do not need to be digested with DpnI or be extensively purified prior to use.

**Figure 1:**
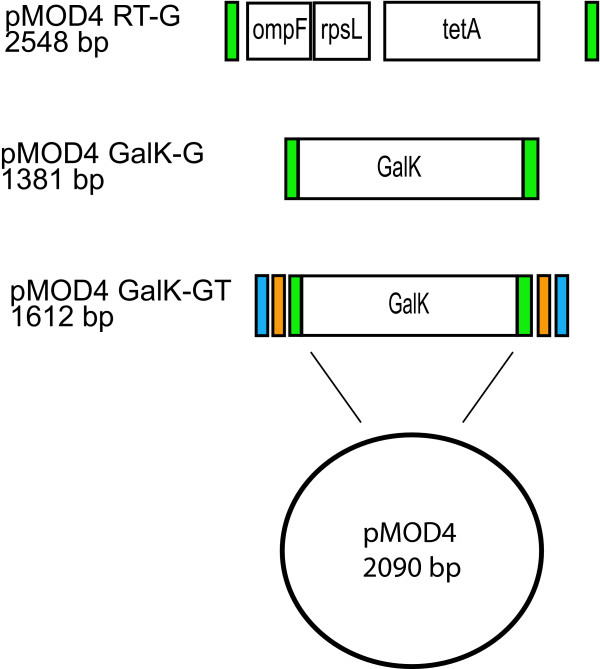
**Diagram of pMOD4-RT-G, pMOD4-*galK*-G, and pMOD4-*galK*-GT**. The pMOD4-RT-G plasmid consists of the RT cassette flanked by 50 nucleotide regions identical to the 5' and 3' ends of FLAG-GFP (green) while pMOD4-*galK*-G consists of the *galK *cassette flanked by the same sequences. pMOD4-*galK*-GT consists of the *galK *cassette flanked by both the FLAG-GFP homology regions and 50 nucleotide regions identical to the 5' and 3' ends of N-terminal and C-terminal TAP (blue and orange, respectively). Both plasmids utilize the R6K-based pMOD4 backbone which is unable to replicate in SW106.

For *C. elegans *experiments most transgenes consist of GFP fusions constructed to either identify the location and timing of gene expression and/or to visualize the cellular localization of the protein. To construct a GFP fusion transgene using the method of Dolphin et. al. two pairs of long oligonucleotides need to be ordered and purified for the generation of a transgene [6]. The first set anneal to the RT cassette and carry 50 base pair homology arms specific for the gene of interest. The second set anneals to GFP and carry the same homology arms. If these are purchased from commercial companies the cost of these oligos is not insignificant especially if multiple transgenes are to be generated. To facilitate the construction of GFP fusion genes, we inserted oligos with 50 base pair homology arms for GFP into pMOD4 RT to generate pMOD4 RT-G (Figure 1, Additional files 1, 2, and 3). The presence of these homology arms allows a single set of oligos to be used for the amplification of the RT cassette and GFP (Figure 1). This reduces the cost for oligos by half compared with the prior technique.

During the construction of pMOD4 RT and *daf-12 *transgenes using the RT cassette we found two limitations of the RT cassette. First, the RT cassette appears to be somewhat toxic to *E. coli *as pMOD4 RT could only be maintained in the JM109λpir strain where it is a low copy number plasmid instead of the EC100*pir-116 *strain which increases the copy number of R6K based plasmids to ~250. This toxicity may contribute to some of the difficulties we experienced with positive and negative selection of fosmids carrying the cassette. Second, the RT cassette involves separate genes for positive and negative selection during recombineering [12]. This property may increase the background during negative selection as mutations in the gene involved would be silent until the bacteria are plated on selective media [9]. These mutations are most likely introduced during PCR amplification as 10^6 ^amplification of the template would be expected to produce ~2–6% of products with mutations for proofreading polymerases and up to 25% for *Taq*-proofreading polymerase blends [13]. Hence, any toxicity of the cassette would facilitate selection of mutated, less toxic clones. To address these two concerns we experimented with the use of the *galK *gene for positive and negative selection during recombineering [9]. To facilitate the use of *galK*, we transferred the *galK *gene to the pMOD4 plasmid and added GFP homology arms for use during recombineering (Figure 1, Additional files 1, 2, and 3).

**Figure 2:**
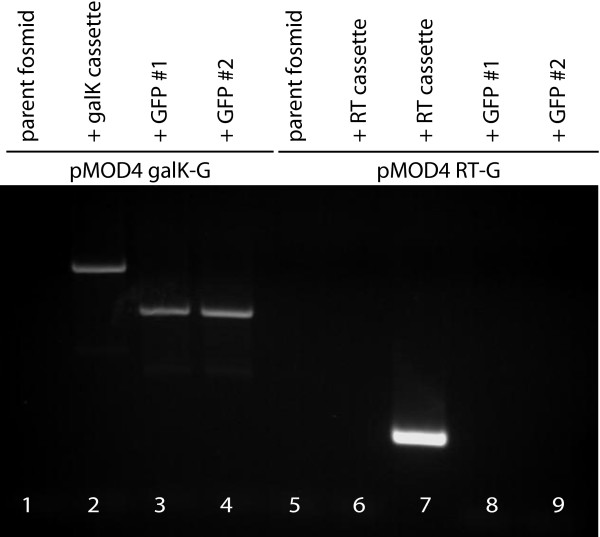
**Production of FLAG-GFP modified fosmids with pMOD4-*galK*-G**. The fosmid carrying the K10C2.4 gene was modified using positive and negative selection using PCR amplified cassettes from the indicated plasmids. The effectiveness of fosmid modification was monitored via PCR using the indicated oligo sets. Lane 1, 2, 3, 4, 5, 6, 8 and 9 FLAG-GFP F and FLAG-GFP R; Lane 7 RT screen F and RT screen R.

We initially compared the use of pMOD4 RT-G and pMOD4 *galK*-G by constructing a GFP fusion at the N-term of the K10C2.4 gene which encodes a member of the fumarylacetoacetate hydrolase family (Figure 2) [14]. Prior attempts to create a GFP reporter using the promoterome library clone were unsuccessful perhaps due to a lack of important promoter elements in the promoter clone (A.F., unpublished data). We found that the RT and *galK *cassettes can be easily inserted into a fosmid carrying K10C2.4 by recombineering (Figure 2 lanes 2 and 7). We then used a PCR fragment carrying FLAG-tagged GFP to replace either the RT or *galK *cassette via a second round of recombineering. Colonies with successful substitution of FLAG-GFP were identified by using selective media. We found the *galK *cassette was more robust than the RT cassette as 4/4 colonies created with the *galK *cassette and 0/4 colonies created with the RT cassette (Figure 2 lanes 3, 4, 8. and 9) carried FLAG-GFP by PCR. PCR analysis of a further 48 clones revealed 40/48 colonies created with the *galK *cassette and 0/48 colonies created with the RT cassette carried FLAG-GFP after selection (Table 1).

**Table 1:** Genotype of colonies isolated after recombineering

**Gene**	**Cassette**	**PCR product electroporated**	**Colonies Obtained**	**GFP + colonies**	**Cassette + colonies**
K10C2.4	*galk*	FLAG-GFP	714	40/48	3/48
K10C2.4	*galk*	None	85	0/48	23/48
K10C2.4	RT	FLAG-GFP	55	0/48	46/48
K10C2.4	RT	None	81	0/48	46/48
*dod-22*	*galk*	FLAG-GFP	N.D.	46/48	N.D.
*dod-22*	RT	FLAG-GFP	N.D.	1/48	N.D.
*myo-3*	*galk*	FLAG-GFP	N.D.	46/47	N.D.
*myo-3*	RT	FLAG-GFP	N.D.	2/48	N.D.
*dod-22* (Frozen 1 year)	*galk*	FLAG-GFP	N.D.	16/48	N.D.
*myo-3* (Frozen 1 year)	*galk*	FLAG-GFP	N.D.	18/48	N.D.

The source of this difference appears to be at least in part due to weaker negative selection from the RT cassette as revealed by analysis of colonies obtained after a no PCR product control electroporation. This control was conducted similarly to the second recombineering step but no FLAG-GFP PCR product was added prior to electroporation. PCR analysis of 48 colonies for the *galK *and RT cassettes revealed that 23/48 *galK *cassette bacteria still had at least part of the cassette whereas 46/48 RT colonies still had a cassette detectable by PCR (Table 1). This suggests that a significant number of bacteria with the RT cassette are still able to grow despite negative selection. Further, addition of the FLAG-GFP PCR product to the electroporation resulted in a noticeable increase in colonies from bacteria with the *galK *cassette but not from bacteria with the RT cassette (Table 1). These counts were performed in parallel and included bacteria from undiluted, 1:10 diluted, and 1:100 diluted samples plated from a single experiment. Together, the greater number of total colonies obtained from bacteria with the *galK *cassette along with the more effective negative selection might lead to the greater effectiveness of the *galK *cassette in recombineering as the significant number of true positives could now outnumber the relatively small number of false positives. Consistent with this, PCR analysis of the *galK *colonies electroporated with the FLAG-GFP PCR product revealed that only 3/48 colonies retained the *galK *cassette whereas 40/48 colonies correctly have the FLAG-GFP tag instead.

**Figure 3:**
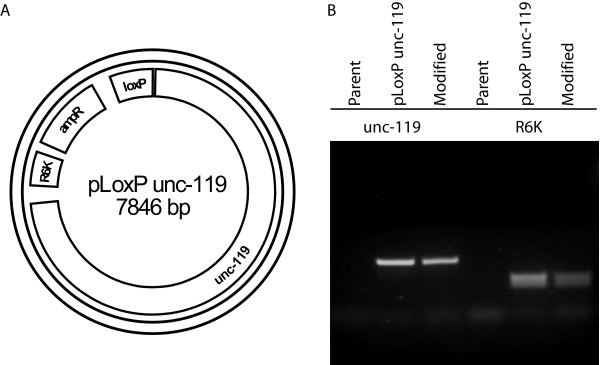
**Addition of a selectable marker with pLoxP *unc-119***. (A) Map of pLoxP *unc-119 *showing the ampicillin resistance gene (ampR), R6K replication origin, and loxP site. (B) Integration of the pLoxP *unc-119 *plasmid into the fosmid carrying K10C2.4 as shown by PCR. The selected ampicillin-resistant colony (modified) carries both the *unc-119 *and R6K sequences from pLoxP *unc-119 *but absent in the parent fosmid which are detected via PCR with the indicated oligo sets. unc-119 unc-119 F and unc-119 R; R6K R6K F and R6K R.

To facilitate the use of modified fosmids for the creation of transgenic animals we exploited the loxP site in the fosmid backbone as a means to quickly insert selectable markers. The SW106 bacterial strain used for recombineering also carries a transgene that expresses the cre recombinase under the control of the arabinose inducible araBAD promoter [9]. We constructed the plasmid pLoxP *unc-119 *which carries the genomic DNA that rescues the *unc-119 *mutant and a loxP site on the pMOD4 vector backbone (Figure 3A, Additional files 1, 2, and 3). This plasmid is unable to replicate in SW106 due to the R6K replication origin. We were able to integrate this plasmid into fosmids in SW106 by using electroporation followed by recovery in LB containing arabinose (Figure 3B and 3C). Fosmids with successful integration were identified by selection on LB plates containing chloramphenicol and ampicillin. The latter antibiotic selects for integration of the ampicillin resistance gene carried on the pMOD4 backbone into the fosmid loxP site. While the cre-lox recombination event is reversible, we have not found this to be a significant problem due to continued selection for ampicillin resistance and the use of the EPI300 bacteria strain, which lacks cre recombinase, for large-scale growth and long-term storage of modified fosmids.

**Figure 4:**
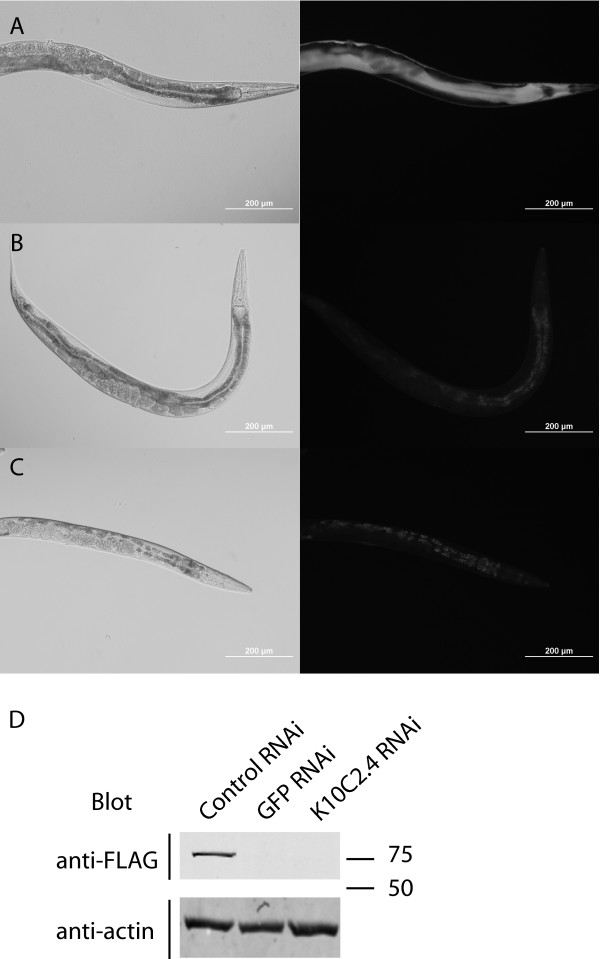
**Generation of transgenic animals with modified fosmids**. The modified fosmid carrying the K10C2.4:FLAG-GFP transgene was introduced into the DP38 (*unc-119(ed3)*) strain via microparticle bombardment. (A) GFP expression seen in the transgenic worms. (B and C) GFP expression is reduced by treatment with GFP (B) or K10C2.4 (C) RNAi. (D) Detection of the FLAG-GFP transgene in RNAi treated worm extracts by western blotting with anti-FLAG antibodies. Equal loading was confirmed by blotting with anti-actin antibodies.

We tested whether the *K10C2.4::GFP *transgene created was functional by creating transgenic animals via biolistic bombardment. We obtained a mix of transgenic animals carrying extrachromosomal arrays and integrated transgenes. Analysis of >15 lines revealed a similar pattern of GFP expression in the intestine and hypodermis (Figure 4A and data not shown). Additionally, the transgene produced a K10C2.4:GFP fusion protein in vivo based on two lines of evidence. First, RNAi directed against either GFP or K10C2.4 resulted in a loss of GFP expression as seen by fluorescence microscopy (Figure 4B and 4C). Second, western blotting using α-FLAG antibodies, which detect a FLAG epitope at the start of GFP, identified a 76 kD protein consistent with a K10C2.4:GFP fusion protein, and RNAi directed at GFP or K10C2.4 result in the loss of this protein (Figure 4D). Together these data indicate the ability of recombineering to produce transgenes capable of producing fusion proteins and to introduce new epitope tags into target genes.

**Figure 5:**
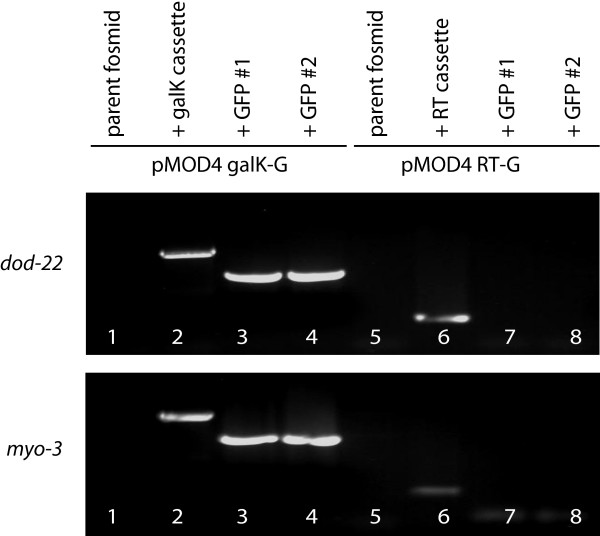
**Comparison of fosmid modification using pMOD4-*galK*-G with pMOD4-RT-G**. Fosmids carrying the *dod-22 *(top) or *myo-3 *genes (bottom) were modified to generate FLAG-GFP fusion genes through positive and negative selection using either the *galK *or RT cassette. The effectiveness of fosmid modification was monitored via PCR using the indicated oligo sets. Lane 1, 2, 3, 4, 7 and 8 FLAG-GFP F and FLAG-GFP R; Lane 5 and 6 RT screen F and RT screen R.

Our work with K10C2.4 suggested that the *galK *cassette was more robust for the negative selection step than the RT cassette. To explore this observation, we created FLAG-GFP fusion genes for the *dod-22 *and *myo-3 *genes. We found that similarly to K10C2.4, the *galK *cassette proved more reliable in head-to-head testing with the RT cassette with regards the isolation of correctly modified fosmids after negative selection (Figure 5, lanes 3 and 4 compared to lanes 7 and 8). With the screening of 48 additional colonies, we found 1/48 *dod-22 *and 2/48 *myo-3 *colonies carrying FLAG-GFP for colonies created with the RT cassette whereas for the *galK *cassette 46/48 *dod-22 *and 46/47 *myo-3 *colonies carried FLAG-GFP (Table 1). Remarkably, we found that colonies carrying FLAG-GFP could also be obtained with induced competent cells stored at -80°C for a full year (Table 1).

**Figure 6:**
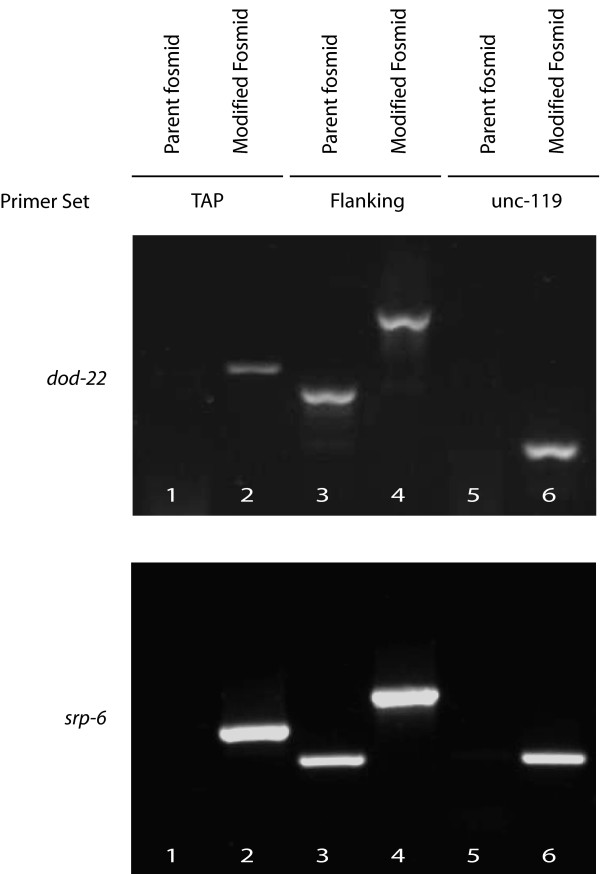
**Generation of TAP modified fosmids with pMOD4-*galK*-GT**. Fosmids carrying *dod-22 *(top) or *srp-6 *genes (bottom) were modified to generate a C-terminal (*dod-22*) and N-terminal (*srp-6*) TAP fusion transgene. After modification, pLoxP *unc-119 *was then integrated into the fosmid backbone. The effectiveness of fosmid modification was monitored via PCR using the indicated oligo sets. Lane 1 and 2 C-term TAP F and C-term TAP R (top) and N-term TAP F and N-term TAP R (bottom); Lane 3 and 4 primers derived from the sequences flanking the insertion site which produce an up-shift following insertion of the tag (sequence available upon request); Lane 5 and 6 unc-119 F and unc-119 R.

Besides the production of GFP fusion proteins, an increasing number of papers in *C. elegans *research are describing the use of transgenic worm strains for proteomic studies aimed at identifying protein interaction partners [15-19]. One approach has been to use the TAP tag which consists of a combination of protein-A and calmodulin binding peptide epitopes which allow the purification of tagged proteins at high purity via the use of two successive purification steps [20]. The TAP tag has been successfully used in *C. elegans *to purify the *unc-29 *acetylcholine receptor subunit along with multiple interacting proteins that were subsequently identified by mass spectrometry [21]. To facilitate the creation of TAP-tagged transgenes we modified pMOD4 *galK*-G to add regions complementary to either the N-terminal or C-terminal version of the TAP tag. The resulting plasmid, pMOD4 *galK*-GT, allows a single set of oligos to be used for the generation of the tagged fosmid through sequential rounds of recombineering (Figure 6).

## Discussion

### A streamlined procedure for transgene production

We describe a modified protocol and accompanying vectors that can be used to easily produce fosmid-based transgenes for the generation of transgenic *C. elegans*. Our protocol involves three steps which are all carried out in the SW106 bacterial stain.

In the first step, the *galK *gene is integrated into the desired site in the fosmid to be modified through recombineering with a PCR product carrying *galK *flanked by homology arms complementary to the target fosmid (Additional files 1, 2, and 3). We have improved this step relative to prior techniques via the integration of homology regions for either FLAG-GFP or a TAP tag into the *galK *cassette. This allows a single set of oligos to be used for the first and second steps of fosmid modification and lowers the cost of fosmid modification by 50%. The integration of GFP homology arms into the RT cassette has also been recently described by Bamps and Hope [5]. This modification is especially important if the technique is to be scaled up to genome-wide coverage. We further simplify the first step through the use of a *galK *cassette carried on an R6K-based vector which is unable to replicate in the SW106 bacterial strain. This improvement eliminates the need to either DpnI digest nor extensively purify the PCR product used for recombineering.

In the second step, the *galK *cassette is replaced with tags such as GFP or TAP which are then fused with the gene of interest on the fosmid (Additional files 1, 2, and 3). The success of this step hinges on the effectiveness of the negative selection strategy used to identify correctly modified fosmids from those that are unmodified. We found that the *galK *cassette was more reliable than the previously described RT cassette in head to head testing. Similar results have also been found by another lab (P. Moroni, personal communication). This may be due to the *galK *gene product serving as the target of both positive and negative selection instead the two separate genes in the RT cassette which are involved in each step [9]. The larger size of the RT cassette also required the use a lower fidelity polymerase, i.e. a Taq/proof-reading polymerase versus a proof-reading polymerase alone, for efficient amplification which may serve as a source of mutations in the cassette that could interfere with the negative selection step. Alternately, we have found that the RT cassette is somewhat toxic to bacteria which may lead to the selection for mutations that decrease the effectiveness of negative selection. In contrast, for the *galK *cassette the positive selection step and subsequent re-streaking on MacConkey agar select for robust *galK *expression before *galK *is used in the negative selection step. Additionally, our addition of the FLAG-GFP or TAP homology regions into the *galK *cassette may also increase the efficiency of recombineering by increasing the length of the homology arms the PCR product to a maximum of 100 bp on each end. However, we have failed to see a qualitative difference between the 100 bp homology arm and a shorter FLAG-GFP only PCR product with 50 bp homology arms when tested (data not shown). It still may be possible that for a subset of genes the 100 bp homology arms might make an important contribution. Finally, it may be possible that subtle changes in the protocol used by other groups may have a significant impact on the success of recombineering with the RT cassette as some groups have reported higher efficiency rates with it [6].

In the third step a selectable marker for the generation of transgenic worms is added to the fosmid (Additional files 1, 2, and 3). This creates a marker in cis and directly linked to the modified fosmid. For the selectable marker, we chose the widely used *unc-119 *marker that is compatible with both microinjection and biolistic bombardment [8]. Bombardment is particularly useful as significant numbers of transgenic lines can be generated by a single trial and even inexperienced personnel can create transgenics (data not shown).

As a result of our modifications, the entire procedure can be accomplished from start to production of a transgenic animal in a few weeks even by members of the lab who have a grasp of lab basic techniques but are not expert in molecular biology or microbiology.

### Applications

Our lab has used this procedure to generate both GFP transgenes to examine gene expression pattern and response to RNAi [14]. We have also used this procedure to generate TAP transgenes for proteomic studies, but these transgenic animals could also be used for chromatin or RNA immunoprecipitation assays as well (Figure 6 and not shown). It might be possible to adapt this technique to liquid culture as means to create transgenes in a high throughput manner. Beyond the intended uses of this technique, we have found that recombineering provides the flexibility to generate alternate vectors at a later time. Specifically, the induced, competent bacteria carrying the *galK *intermediate can be stored for later use even months to a year later (Table 1). Oligos carrying homology arms to the target gene can then be used to quickly add other tags generated by PCR. For example, we have been able to generate a transgene carrying a modified TAP tag which has been reported to produce higher purified protein yields in *Drosophila *to conduct head-to-head tests [22]. Making this change only requires a few days of work to reach the stage of generating new transgenic animals. Finally while our work focused on applications in *C. elegans*, it is likely that some of these reagents can be used in the construction of modified BACs or fosmids for other experimental systems.

### Problems encountered

During construction of modified fosmids, we encountered problems with the modification of *srp-6 *and *dod-22*. These fosmids are similar in that both contain multiple genes that are highly similar at the sequence level. For example, the WRM0627aH02 fosmid contains both *srp-5 *and *srp-6 *and the WRM066cB09 fosmid contains *dod-22 *and K10D11.2. For *srp-6 *we discovered a rearrangement during the initial transformation into SW106. This was easily detected via PCR using primers that anneal 50–100 nucleotides away on the genomic sequence flanking the desired modification site. For *dod-22 *we found that the *galK *cassette integrated into another site on the fosmid. This was similarly detected by PCR using flanking oligos. Both of these difficulties could be overcome by diligence during the screening of isolated colonies and are likely due to the presence of highly similar sequences that could lead to spurious recombination events. To guard against moving forward with an incorrect fosmid, we have routinely screened colonies with both cassette-specific and flank sequence-specific oligos to ensure the right insert and right site. The flanking oligos are also useful for the sequencing of the resulting fosmids.

## Conclusion

We have modified existing recombineering protocols to create a simpler and more robust method of generating transgenes for use in the construction of transgenic worms. The approach and vectors will be of benefit especially to smaller academic labs looking to begin to use fosmid-based transgenes.

## Methods

### Strains

Recombineering in *E. coli *was performed using the SW106 (*mcrA *Δ(*mrr-hsdRMS-mcrBC*) Δ*lacX74 deoR endA1 araD139 *Δ(*ara*, *leu*) *7697 rpsL recA1 nupG *φ80d*lacZΔM15 *[λ*c*1857 (*cro-bioA*)<>*Tet*] (*cro-bioA*)<>*araC*-P_BAD _*Cre *Δ*galK*) strain which carries the λred homologous recombination genes under the control of a temperature sensitive λ repressor as well as an arabinose inducible cre recombinase (Table 2) [9]. Fosmids were grown before and after recombineering in the EPI300 (*F*^-^*mcrA Δ(mrr-hsdRMS-mcrBC) φ80dlacZΔM15 ΔlacX74 recA1 endA1 araD139 Δ(ara, leu)7697 galU galK λ*^-^* rpsL nupG trfA tonA*) strain which allows the fosmid copy number to be regulated via the addition of a chemical inducer (Epicentre Biotechnologies, Madison, WI) (Table 2). Plasmids derived from pMOD4 (Epicentre Biotechnologies, Madison, WI) were grown in the EC100D *pir-116 *(F^- ^*mcr*A Δ(*mrr-hsd*RMS-*mcr*BC) φ80d *lac*ZΔM15 Δ*lac*X74 *rec*A1 *end*A1 *ara*D139 Δ(*ara, leu*)7697 *gal*U *gal*K λ^- ^*rps*L *nup*G *pir*-116(DHFR)) (Epicentre Biotechnologies, Madison, WI) or JM109λpir (F' *traD36 proA*^+^*B*^+^*lacI*^*q *^*Δ(lacZ)M15/Δ(lac-proAB) glnV44 e14*^-^*gyrA96 recA1 relA1 endA1 thi hsdR17 λpir*) strains due to the R6K origin which requires the *pir *protein for replication (Table 2) [10]. Transgenic nematodes were created using the DP38 (*unc-119(ed3) III*) strain obtained from the Caenorhabditis Genetics Center which is supported by the NIH.

**Table 2:** Strain and vector availability

**Plasmids**	**Source**	**Available at**
Fosmid clone	Geneservice Ltd.	Geneservice
pGalK	[9]	NCI-Frederick
pMOD4-RT-G	This work	Addgene
pMOD4-*galK*-G	This work	Addgene
pMOD4-*galK*-GT	This work	Addgene
pLoxP- *unc-119*	This work	Addgene
pMOD4-GFP	This work	Addgene
**Bacteria**		
SW106	[9]	NCI-Frederick
EPI300	Epicentre Biotechnologies	Epicentre
EC100D *pir-116*	Epicentre Biotechnologies	Epicentre
JM109λpir	[10]	Cloning Vector Collection

### Plasmids

pBAC-RT was obtained from Dr. Colin Dolphin [12]. pMOD4-RT was generated by digesting pBAC-RT with EcoRI and ApaI and ligating this fragment into pLoxP. The resulting plasmid was digested with XbaI and EcoRI and the fragment carrying the RT cassette was ligated into pMOD4 (Epicentre Biotechnologies, Madison, WI) (Table 2). pMOD4-RT could only propagated in JM109λpir where the plasmid is low copy number. pMOD4-RT-G was created from pMOD4-RT by insertion of oligos containing 50 bp regions of homology to the N and C-terminal ends of FLAG-GFP (sequence available on request). This resulted in an RT cassette that was slightly larger than that generated via PCR by Dolphin et. al. [6]. pMOD4 *galK*-G was constructed from pGalK (a gift of Drs. Soren Warming and Neal Copeland) by insertion of oligos containing 50 bp regions of homology to the N and C-terminal ends of FLAG-GFP into the plasmid (sequence available on request). The oligos and *galK *cassette were then removed as an XbaI-Acc65I fragment and ligated into pMOD4 digested with XbaI and Acc65I. pMOD4 *galK*-GT was constructed from pMOD4 *galK*-G by insertion of oligos containing 50 bp regions of homology to the N and C-terminal ends of the N and C-terminal TAP tags from the plasmids pBS1761 and pBS1479, respectively (Table 2) [20,23]. pMOD4 GFP was generated by cloning a PstI-EcoRI fragment from pFLAG-GFP Daf-12 into pMOD4 digested with PstI and EcoRI. This fragment contains a 5' FLAG epitope fused to GFP from pPD117.01 (a gift from Dr. Andy Fire) and the N-terminal 328 amino acids of *daf-12 *(Table 2). pLoxP *unc-119 *was generated by inserting an oligo containing a loxP site into pMOD4 (sequence available on request) and digesting the resulting plasmid with XbaI and ApaI. The *unc-119 *gene was excised from pDP#MM016b as an XbaI-ApaI fragment and inserted into pLoxP (Table 2) [24].

The following fosmids were obtained from Geneservice Ltd. (Cambridge, UK): WRM066cB09 – *dod-22*, WRM0615cC09 – K10C2.4, WRM061015 – *myo-3*, WRM0627aH02 – *srp-6 *(Table 2).

### Recombineering

Electrocompetent SW106 cells were made by growing a 5 mL overnight culture in LB broth with chloramphenicol (12.5 μg/mL) (Additional files 1, 2, and 3). 1 mL was then inoculated into 100 mL of LB with chloramphenicol in a 2 L flask and grown to an OD_600 _0.6–0.8. 50 mL of the culture was then transferred to a sterile 250-mL flask and induced by gently shaking (100 rpm) in a water bath at 42°C for 20 minutes. In control experiments we found that use of a shaking water bath was critical for effective induction. These can be often purchased used for much less than new equipment. The non-induced control was shaken gently for the same amount of time at 32°C, and then both cultures were incubated on ice for 15 minutes. Each culture was then pelleted and washed twice in 50 mL ice-cold 10% glycerol. All but ~500 μl of each supernatant was aspirated and the pellets were resuspended by gentle vortexing. 100 μl aliquots were then stored at -80°C until use.

Electroporation was performed using PCR fragments generated from pMOD4 RT-G, pMOD4 *galK*-G, or pMOD4 *galK*-GT using an Eppendorf 2510 electroporator (Eppendorf, Westbury, NY) at 1350 volts in 0.1 cm gap cuvettes. Oligos for PCR consisted of the FLAG-GFP or TAP sequences fused 3' to 50 bp homology arms derived from the desired site of integration in the target gene (Table 3). PCR products were routinely gel purified but not DpnI digested. For the RT cassette, 150 ng of PCR product was used and the cells were recovered in 1 mL of SOB [-MG] for 1–3 hours before washing twice in M9 buffer. At the end, the induced cells were brought up in 1 mL and the non-induced cells in 0.5 mL M9. Serial dilutions of 1:1, 1:10, and 1:100 were plated on LB plates with tetracycline (5 μg/m) and chloramphenicol (12.5 μg/mL). For the *galK *cassette a similar protocol was followed, except after electroporation the cells were recovered in LB for 4.5 hours and they were plated on MOPS minimal media (Teknova Inc., Hollister, CA) supplemented with 0.2% galactose, leucine (45 mg/L), biotin (1 mg/L), and Cm (12.5 μg/mL). The plates were incubated at 32°C for 3–4 days and then 4 colonies for RT and *galK *cassettes were each streaked onto new plates, for RT the same type of plates were used, while *galK *was streaked onto MacConkey agar plates containing galactose and chloramphenicol (12.5 μg/mL). These plates were incubated at 32°C for 3 days. 4 colonies of each were picked and used to make 5 mL liquid cultures in LB with tetracycline (5 μg/mL) for RT and chloramphenicol (12.5 μg/mL) for both. 0.5 μL of each overnight culture was used for PCR to confirm insertion of the cassette.

**Table 3:** Oligonucleotides used for PCR

**Oligo**	**Sequence**
C-term TAP F	ATGGAAAAGAGAAGATGGAAAAAG
C-term TAP R	GGTTGACTTCCCCGC
FLAG-GFP F	ATGGATTACAAGGACGATGACGATAAGATGAG
FLAG-GFP R	CAAAGCTTGTGGGCTTTTGTATAG
N-term TAP F	ATGGCAGGCCTTGCGC
N-term TAP R	AAGTGCCCCGGAGGATGAGATTTTCT
R6K F	CCTTAGAGGCTATTTAAGTTGCTG
R6K R	GTACTAAGCTCTCATGTTTCAC
RT screen F	ACGTTAACCGGGCTGCAT
RT screen R	GCCGTCAATAAGTTCTGTCAA
unc-119 F	CAAATCCGTGACCTCGACAC
unc-119 R	CACAGTTGTTTCTCGAATTTGG

For the second recombineering process, PCR-generated fragments from pMOD4 FLAG-GFP, pBS1761 (N-term TAP), or pBS1479 (C-term TAP) were generated using the same oligos used in the first round or using shorter GFP or TAP-specific oligos (Table 3). The products (100 ng.) were electroporated into induced competent SW106 cells prepared as above. The electroporation was done as above, except bacteria containing the RT cassette were recovered in SOC and plated on NSLB agar plates containing chloramphenicol (12.5 μg/mL) and streptomycin (500 μg/mL) and those containing the *galK *cassette on MOPS minimal media plates containing 0.2% 2-deoxy-galactose (DOG) and 0.2% glycerol in addition to leucine (45 mg/L), biotin (1 mg/L), and chloramphenicol (12.5 μg/mL) [12]. The plates were incubated for 3–5 days. While we followed relative colony counts on the induced and un-induced plates, we found that there was not always a direct correlation between the counts and successfully identifying clones with correctly modified fosmids. 4 colonies were used to make 5 mL overnight cultures in LB with chloramphenicol (12.5 μg/mL) and these were used for colony PCR as above to confirm that the cassette was inserted. DNA sequencing using flanking oligos was also used to verify correct modification of the fosmid.

### Addition of the unc-119 selectable marker

To facilitate the identification of transgenic worms following biolistic transformation, we inserted the *unc-119 *gene into the fosmid backbone via cre-lox mediated recombination using the pLoxP *unc-119 *plasmid (Additional files 1, 2, and 3) [11,24]. pLoxP *unc-119 *carries the *unc-119 *gene on a R6K-based suicide vector that is unable to replicate in the SW106 bacteria (data not shown). Recombination was accomplished by preparing competent SW106 bacteria carrying the modified fosmid followed by electroporation with 50 ng. pLoxP *unc-119 *as described above. Following electroporation, bacteria were allowed to recover in LB containing 0.1% arabinose for 1 hour. Aliquots were plated on LB plates containing ampicillin (50 μg/mL) and chloramphenicol (12.5 μg/mL) which selects for integration of pLoxP into the fosmid. The presence of the *unc-119 *gene could also be verified via PCR (Table 3 and Figure 3).

### Generation of transgenic worms

Fosmids modified through two rounds of recombineering and addition of the *unc-119 *gene were isolated via mini-prep using the FosmidMAX DNA purification kit (Epicentre Biotechnologies, Madison, WI). This DNA was used to electroporate the EPI300 bacterial strain which allows library fosmids to be amplified by following the manufacturer's instructions (Epicentre Biotechnologies, Madison, WI). Amplified fosmids were purified from 50 mL of culture using the same kit. We also used a Qiagen maxi-prep kit (Qiagen Inc., Valencia, CA) but found significant amounts of sheared DNA compared with the FosmidMAX kit (not shown). 10 ug of fosmid DNA was used to transform the DP38 *unc-119(ed3) *mutant strain via gold microparticle bombardment using a published protocol [25]. Transgenic animals were identified via the rescue of the *unc-119 *mutation by the rescue fragment carried on the fosmid.

RNAi treatment was conducted using the feeding approach. The K10C2.4 and GFP RNAi constructs have been previously described [14,26]. Photomicrographs were taken using an Olympus BX51 upright microscope fitted with FITC filters for detection of GFP. All photos were taken on the same day using the same camera settings to allow direct comparison of images. Western blotting was performed on worm extracts as previously described [14]. Detection was performed using the anti-FLAG antibody (Sigma-Aldrich, St. Louis, MO) followed by incubation with an IR dye 800 donkey anti-mouse secondary antibody (Li-Cor Biosciences, Lincoln, NE). Equal loading was confirmed by use of an anti-actin antibody as previously described [14].

## Authors' contributions

YZ conceived of the study, constructed plasmids, conducted recombineering experiments, participated in data analysis, and contributed to writing and editing the manuscript. LN constructed plasmids, conducted recombineering experiments, and contributed to writing and editing the manuscript. ALF conceived of the study, participated in data analysis, and wrote the manuscript.

## Supplementary Material

Additional file 1 **Legend for overview of recombineering procedures.** Figure legend for Additional files 2 and 3. Click here for file

Additional file 2 **Merged overview of recombineering procedures.** A merged figure showing the steps and time involved in recombineering using the original RT cassette, modified RT cassette, and *galK *cassette. Click here for file

Additional file 3 **Separate overviews of recombineering procedures.** Separate figures showing the steps and time involved in recombineering using the original RT cassette, modified RT cassette, and *galK *cassette. These are the same figures that are merged in Additional file 2, but provided separately for ease of reading or printing. Click here for file
