# Supplementary material for: A simplified, robust, and streamlined procedure for the production of C. elegans transgenes via recombineering
Source: BMC Dev Biol. 2008 Dec 30;8:119. doi: 10.1186/1471-213X-8-119 (PMC2629773; doi:10.1186/1471-213X-8-119)
Supplement: Additional file 1 — Legend for overview of recombineering procedures. Figure legend for Additional files 2 and 3. [file 1471-213X-8-119-S1.doc]

**Additional Files 2 and 3 – Overview of recombineering process**

Additional file 2 shows a merged figure comparing the recombineering vectors and procedures. (Upper left) Schematic diagrams of vectors pMOD4 *galK*-G, pMOD4 *galK*-GT, pMOD4 RT-G, pMOD4 GFP, and pLoxP *unc-119*. (Upper right) Outline of recombineering procedure as described by Dolphin et. al. including time required for each step [6]. A fosmid of interest is modified in a two-step procedure involving the insertion of the RT cassette followed by replacement of this cassette by GFP or other sequences. (Lower right) Outline of modified RT recombineering procedure as described in methods including time required for each step. A fosmid of interest is first modified in a two-step procedure involving the insertion of the RT cassette flanked by 50 bp regions of homology to FLAG-GFP followed by replacement of this cassette by FLAG-GFP. Later the *unc-119* marker for use in generating transgenic animals is inserted into the loxP site on the fosmid backbone. (Lower left) Outline of *galK* recombineering procedure as described in methods including time required for each step. A fosmid of interest is first modified in a two-step procedure involving the insertion of the *galK* cassette flanked by 50 bp regions of homology to FLAG-GFP or TAP followed by replacement of this cassette by FLAG-GFP or TAP. Later the *unc-119* marker for use in generating transgenic animals is inserted into the loxP site on the fosmid backbone. This overview is also provided as separate figures to facilitate reading, printing, or side-by-side comparison in Additional file 3.
