# Supplementary material for: A simplified, robust, and streamlined procedure for the production of C. elegans transgenes via recombineering
Source: BMC Dev Biol. 2008 Dec 30;8:119. doi: 10.1186/1471-213X-8-119 (PMC2629773; doi:10.1186/1471-213X-8-119)
Supplement: Additional file 2 — Merged overview of recombineering procedures. A merged figure showing the steps and time involved in recombineering using the original RT cassette, modified RT cassette, and galK cassette. [file 1471-213X-8-119-S2.ppt]

## Slide 1
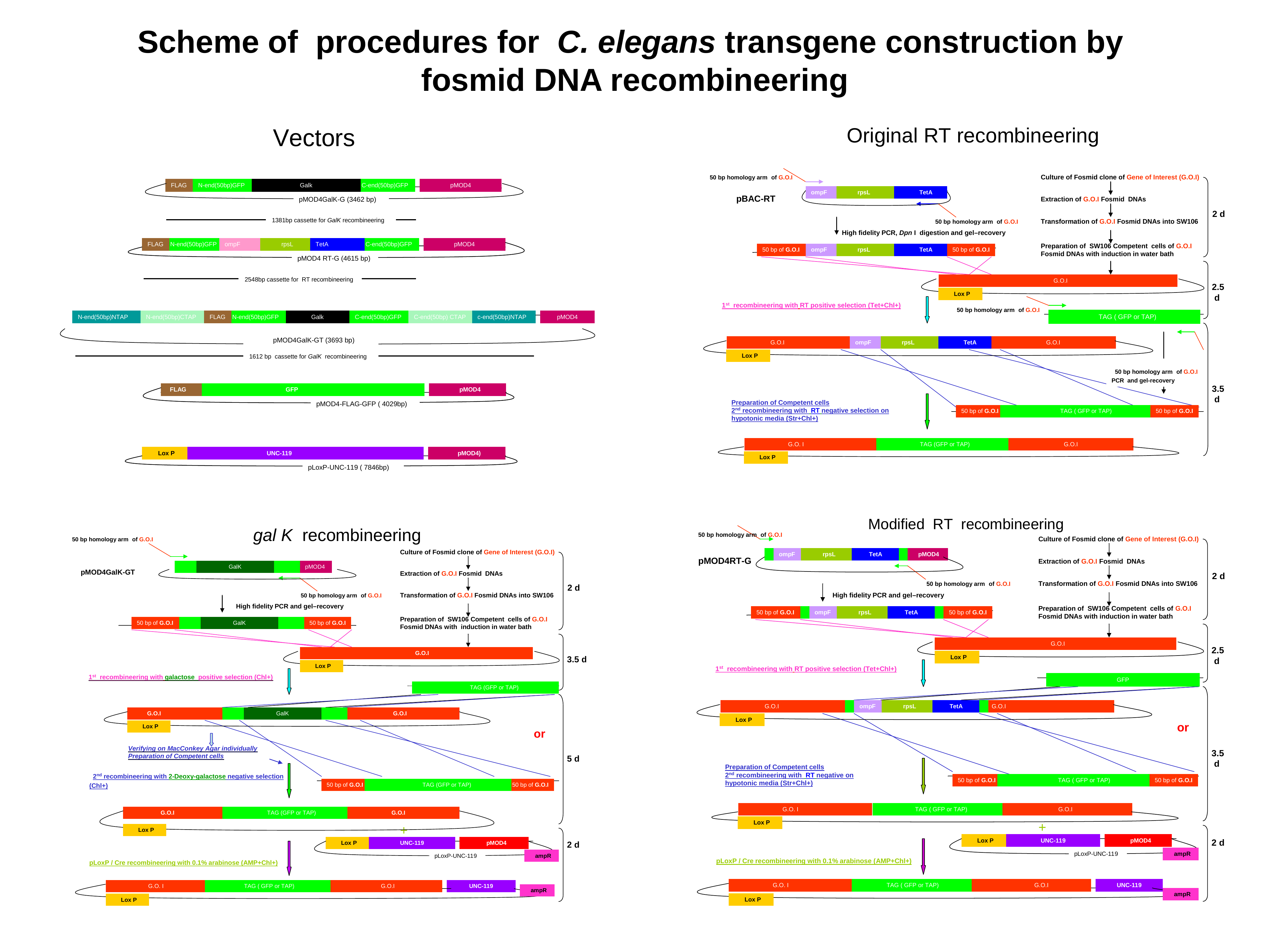

# Scheme of procedures for C. elegans transgene construction by fosmid DNA recombineering
