# Supplementary material for: A simplified, robust, and streamlined procedure for the production of C. elegans transgenes via recombineering
Source: BMC Dev Biol. 2008 Dec 30;8:119. doi: 10.1186/1471-213X-8-119 (PMC2629773; doi:10.1186/1471-213X-8-119)
Supplement: Additional file 3 — Separate overviews of recombineering procedures. Separate figures showing the steps and time involved in recombineering using the original RT cassette, modified RT cassette, and galK cassette. These are the same figures that are merged in Additional file 2, but provided separately for ease of reading or printing. [file 1471-213X-8-119-S3.ppt]

## Slide 1
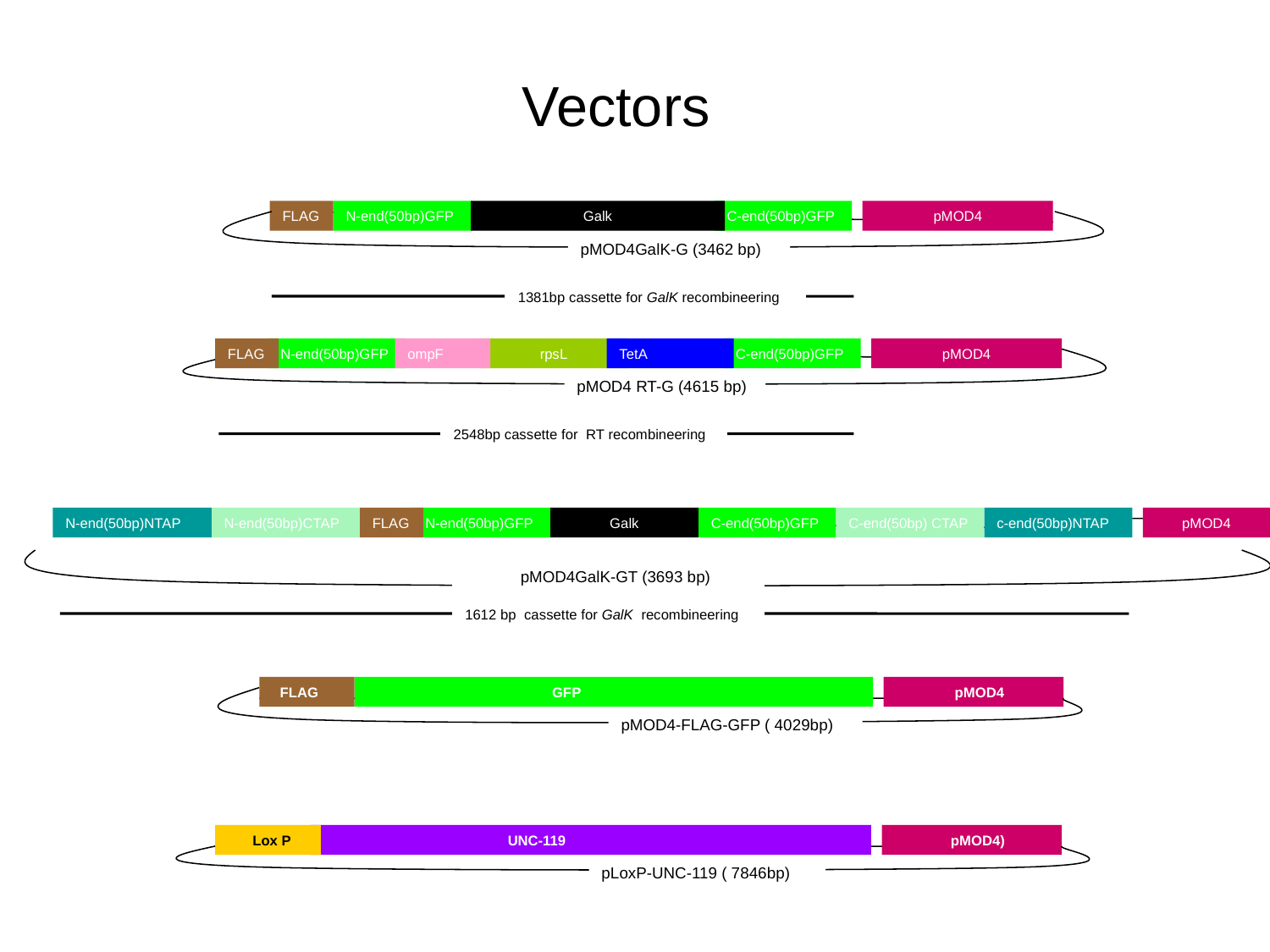

Vectors
FLAG
N-end(50bp)GFP
Galk
C-end(50bp)GFP
pMOD4
pMOD4GalK-G (3462 bp)
1381bp cassette for GalK recombineering
FLAG
N-end(50bp)GFP
ompF
rpsL
TetA
C-end(50bp)GFP
pMOD4
pMOD4 RT-G (4615 bp)
2548bp cassette for RT recombineering
N-end(50bp)NTAP
N-end(50bp)CTAP
FLAG
N-end(50bp)GFP
Galk
C-end(50bp)GFP
C-end(50bp) CTAP
c-end(50bp)NTAP
pMOD4
pMOD4GalK-GT (3693 bp)
1612 bp cassette for GalK recombineering
 FLAG
 GFP
 pMOD4
pMOD4-FLAG-GFP ( 4029bp)
 Lox P
 UNC-119
 pMOD4)
pLoxP-UNC-119 ( 7846bp)

## Slide 2
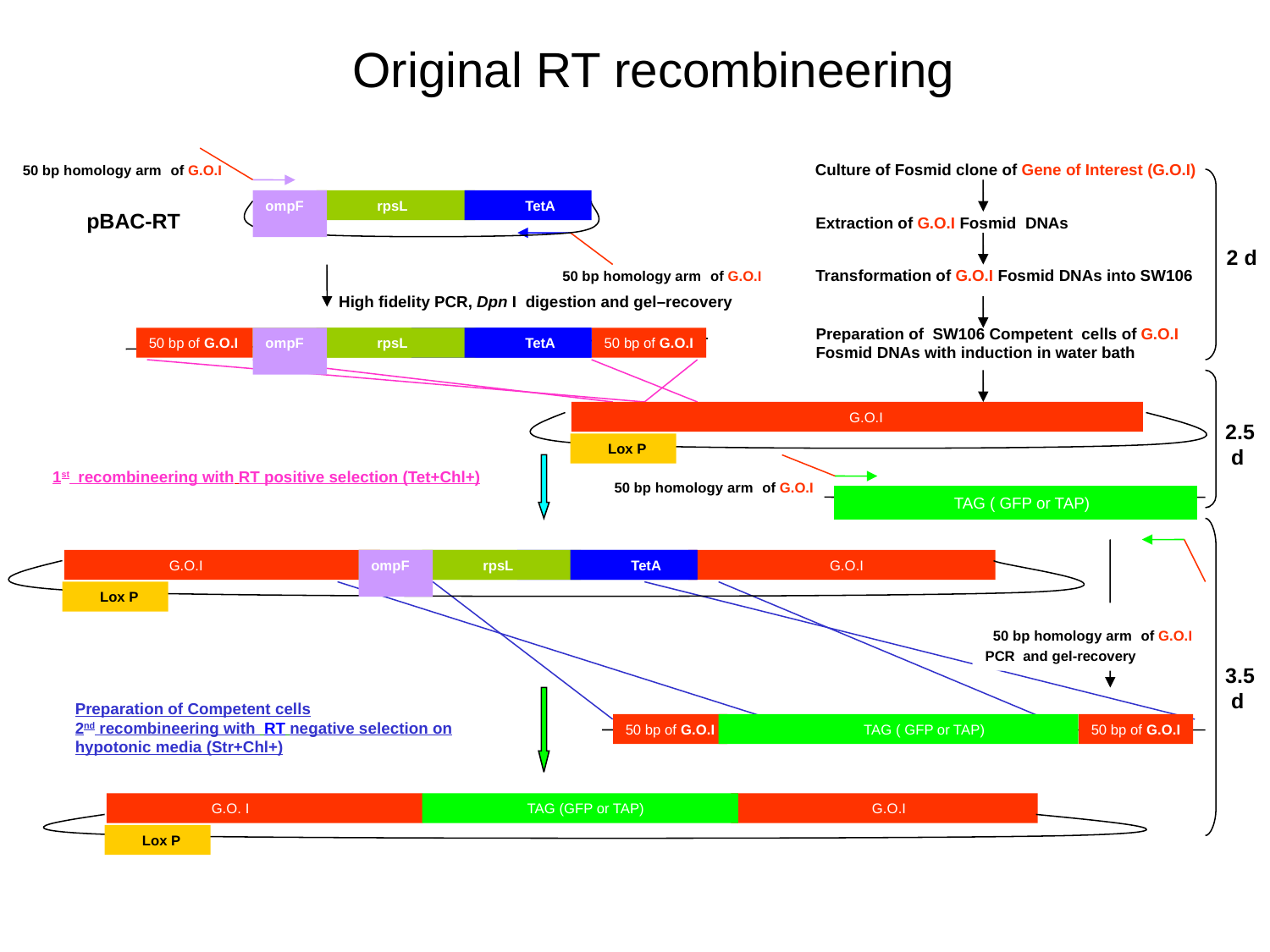

Original RT recombineering
50 bp homology arm of G.O.I
Culture of Fosmid clone of Gene of Interest (G.O.I)
ompF
 rpsL
 TetA
Extraction of G.O.I Fosmid DNAs
pBAC-RT
2 d
50 bp homology arm of G.O.I
Transformation of G.O.I Fosmid DNAs into SW106
High fidelity PCR, Dpn I digestion and gel–recovery
Preparation of SW106 Competent cells of G.O.I
Fosmid DNAs with induction in water bath
50 bp of G.O.I
 rpsL
 TetA
ompF
 rpsL
 TetA
50 bp of G.O.I
 G.O.I
2.5
 d
 Lox P
 1st recombineering with RT positive selection (Tet+Chl+)
50 bp homology arm of G.O.I
 TAG ( GFP or TAP)
 G.O.I
 rpsL
ompF
 rpsL
 TetA
 TetA
 G.O.I
 Lox P
 50 bp homology arm of G.O.I
PCR and gel-recovery
3.5
 d
Preparation of Competent cells
2nd recombineering with RT negative selection on
hypotonic media (Str+Chl+)
50 bp of G.O.I
 TAG ( GFP or TAP)
50 bp of G.O.I
 G.O. I
 TAG (GFP or TAP)
 G.O.I
 Lox P

## Slide 3
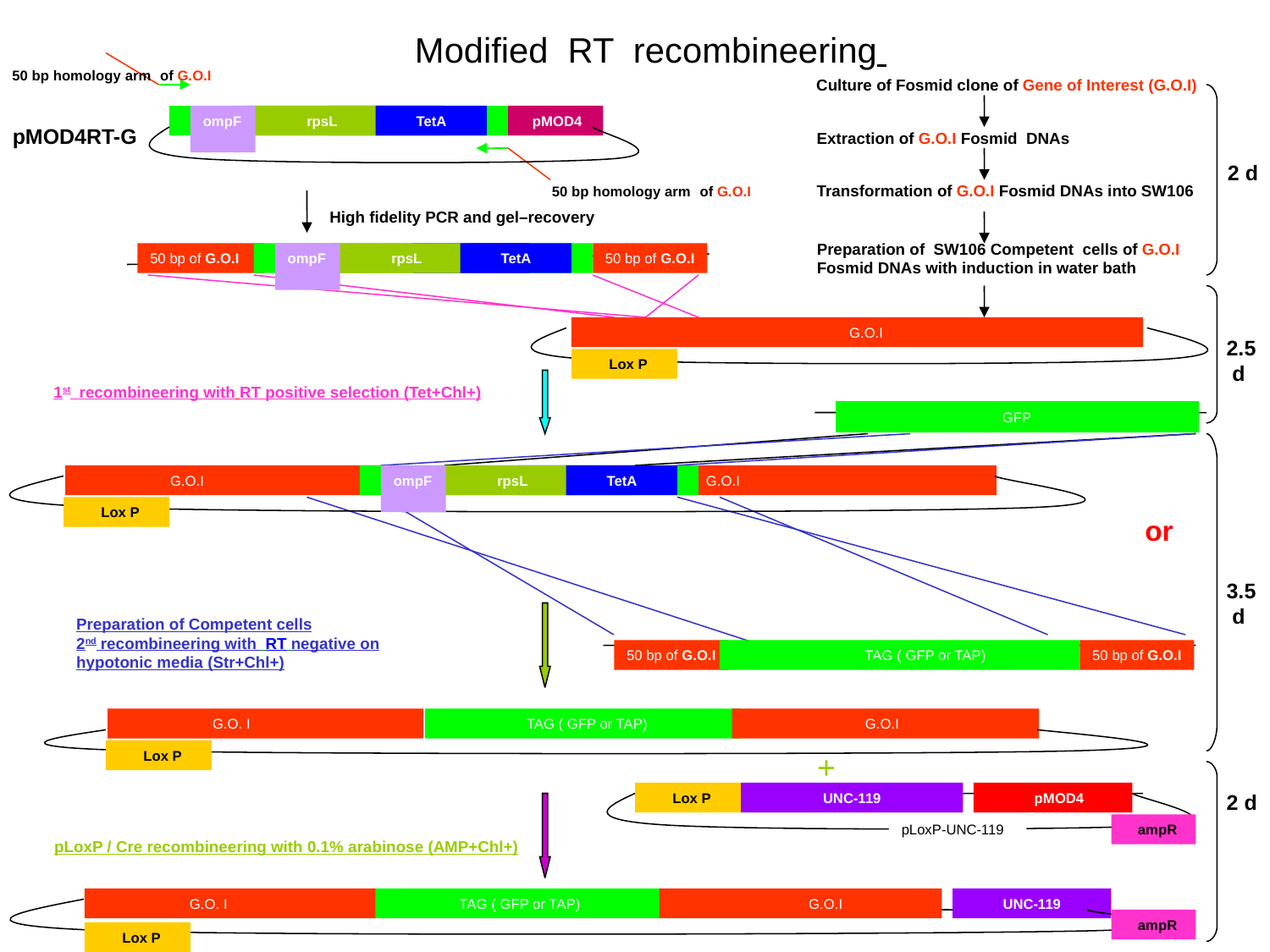

Modified RT recombineering
50 bp homology arm of G.O.I
Culture of Fosmid clone of Gene of Interest (G.O.I)
rpsL
TetA
ompF
 rpsL
 TetA
 pMOD4
Extraction of G.O.I Fosmid DNAs
pMOD4RT-G
2 d
50 bp homology arm of G.O.I
Transformation of G.O.I Fosmid DNAs into SW106
High fidelity PCR and gel–recovery
Preparation of SW106 Competent cells of G.O.I
Fosmid DNAs with induction in water bath
50 bp of G.O.I
rpsL
TetA
ompF
 rpsL
 TetA
50 bp of G.O.I
 G.O.I
2.5
 d
 Lox P
 1st recombineering with RT positive selection (Tet+Chl+)
GFP
 G.O.I
rpsL
TetA
ompF
 rpsL
 TetA
 G.O.I
 Lox P
or
3.5
 d
Preparation of Competent cells
2nd recombineering with RT negative on
hypotonic media (Str+Chl+)
50 bp of G.O.I
 TAG ( GFP or TAP)
50 bp of G.O.I
 G.O. I
 TAG ( GFP or TAP)
 G.O.I
 Lox P
+
 Lox P
UNC-119
 pMOD4
2 d
pLoxP / Cre recombineering with 0.1% arabinose (AMP+Chl+)
pLoxP-UNC-119
 ampR
 G.O. I
 TAG ( GFP or TAP)
 G.O.I
UNC-119
 ampR
 Lox P

## Slide 4
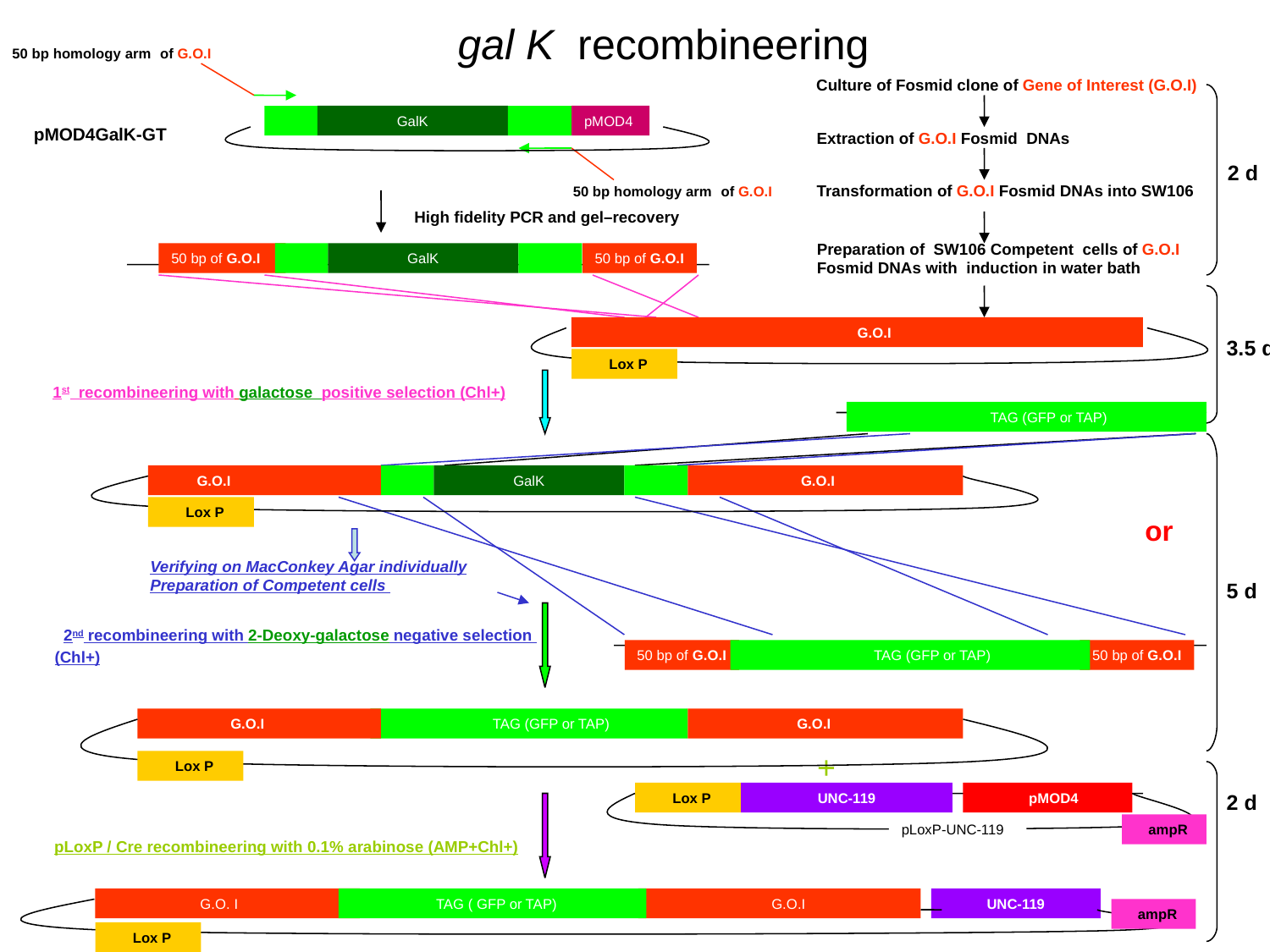

gal K recombineering
50 bp homology arm of G.O.I
Culture of Fosmid clone of Gene of Interest (G.O.I)
GalK
pMOD4
Extraction of G.O.I Fosmid DNAs
pMOD4GalK-GT
2 d
50 bp homology arm of G.O.I
Transformation of G.O.I Fosmid DNAs into SW106
High fidelity PCR and gel–recovery
Preparation of SW106 Competent cells of G.O.I
Fosmid DNAs with induction in water bath
50 bp of G.O.I
GalK
50 bp of G.O.I
 G.O.I
3.5 d
 Lox P
 1st recombineering with galactose positive selection (Chl+)
 TAG (GFP or TAP)
 G.O.I
GalK
 G.O.I
 Lox P
or
Verifying on MacConkey Agar individually
Preparation of Competent cells
5 d
 2nd recombineering with 2-Deoxy-galactose negative selection
(Chl+)
50 bp of G.O.I
 TAG (GFP or TAP)
50 bp of G.O.I
 G.O.I
 TAG (GFP or TAP)
 G.O.I
+
 Lox P
 Lox P
UNC-119
 pMOD4
2 d
pLoxP / Cre recombineering with 0.1% arabinose (AMP+Chl+)
pLoxP-UNC-119
 ampR
 G.O. I
 TAG ( GFP or TAP)
 G.O.I
UNC-119
 ampR
 Lox P
